# Impact of 10-valent Pneumococcal Conjugate Vaccine (PCV10) on nasopharyngeal carriage in children 2 years of age: Data from a four-year time series cross-sectional study from Pakistan

**DOI:** 10.1016/j.dib.2021.106828

**Published:** 2021-02-03

**Authors:** Muhammad Imran Nisar, Shahira Shahid, Sajid Muhammad, Farah Khalid, Amjad Hussain, Sheraz Ahmed, Sadia Shakoor, Furqan Kabir, Aneeta Hotwani, Asad Ali, Anita KM Zaidi, Saad B Omer, Fyezah Jehan, Najeeha Iqbal

**Affiliations:** a Department of Pediatrics and Child Health, Aga Khan University, Karachi, Pakistan; b Department of Pathology and Laboratory Medicine, Aga Khan University, Karachi, Pakistan; c Bill & Melinda Gates Foundation, Seattle, WA, USA; d Yale Institute for Global Health, New Haven, CT, USA

**Keywords:** *Streptococcus pneumoniae*, Pneumococcal Vaccines, Herd Immunity, Nasopharyngeal carriage, Surveillance, Pakistan

## Abstract

The dataset described in this paper was collected for a time-series cross-sectional study exploring the impact of 10-valent Pneumococcal Conjugate Vaccine (PCV10) on nasopharyngeal (NP) carriage in children under 2 years of age from a rural population in Sindh, Pakistan. The study was carried out in two union councils of Matiari - Khyber and Shah Alam Shah Jee Wasi (Latitude 25.680298 / Longitude 68.502711). Data was collected on socio-demographics, clinical characteristics and vaccination status using android phone-based application. NP samples were collected using standard World Health Organisation (WHO) techniques, culture and serotyping was done using sequential Multiplex PCR described by Centre for Disease Control, USA. We looked at the carriage rate of vaccine type (VT) and non-vaccine type (NVT) serotypes over time in vaccinated and unvaccinated children. We additionally looked at the predictors for pneumococcal carriage. The uploaded dataset, available on Mendeley data repository (Nisar, Muhammad Imran (2021), “Impact of PCV10 on nasopharyngeal carriage in children in Pakistan”, Mendeley Data, V1, doi:10.17632/t79h6g97gr.1), has 3140 observations in CSV format. Additional files uploaded include a data dictionary and the set of questionnaires. The dataset and accompanying files can be used by other interested researchers to replicate our analysis, carry similar analysis under varying set of assumptions or perform additional exploratory or metanalysis

## Specifications Table

**Table:** 

Subject	Health and medical sciences
Specific subject area	Vaccine surveillance studies, carriage survey
Type of data	Questionnaires, CSV files and data dictionary
How data were acquired	Data was collected by trained study personnel on a custom-built application for android phones. The questionnaires were designed in .xml format using Open Data Kit (ODK) software. Microbiological data was acquired by performing culture and sequential Multiplex PCR assay on the collected Nasopharyngeal swabs. Epidemiological data was then merged with the microbiological data using the common link ID (the study ID). Analysis and visualization were done using STATA version 15.0. and Microsoft Excel.
Data format	The uploaded files on Mendeley consist of 1. “Raw_dataset.csv” contains raw dataset in CSV format on epidemiological and microbiological fields on 3140 children under 2 years of age from a rural population in Pakistan. The data was collected between Oct 2014 to Sep 2018. 2. “Codebook.xls” describing individual fields in the dataset along with the codebook. 3. “DIB_questionaire.docx” is the field epidemiology questionnaire 4. “Lab_questionnaire.docx” is the Lab questionnaire
Parameters for data collection	Children under 2 years of age residing in two union councils (Khyber and Shah Alam Shah Jee Wasi) of Matiari, Sindh. Fifteen age-eligible children were enrolled every week from a continually updated line listing.
Description of data collection	Data was collected during household visits by trained study personnel. Data on socio-demographic and clinical characteristics was collected using questionnaires on smart phones whereas nasopharyngeal specimens were collected using standardised methods for further microbiological analysis.
Data source location	Institution: The Aga Khan University City: Karachi Country: Pakistan Latitude and longitude for collected samples/data: Matiari, Sindh, Pakistan, 25.680298, 68.502711
Data accessibility	The dataset can be accessed through the following link: Nisar, Muhammad Imran (2021), “Impact of PCV10 on nasopharyngeal carriage in children in Pakistan”, Mendeley Data, V1, doi:10.17632/t79h6g97gr.1
Related research article	[1] Nisar MI, Ahmed S, Jehan F, Shahid S, Shakoor S, Kabir F, Hotwani A, Munir S, Muhammad S, Khalid F, Althouse B, Hu H, Whitney C, Ali A, Zaidi AKM, Omer SB, Iqbal N. Direct and indirect effect of 10 valent pneumococcal vaccine on nasopharyngeal carriage in children under 2 years of age in Matiari, Pakistan. Vaccine. 2021 Jan 6:S0264-410X(20)31662-5. doi:10.1016/j.vaccine.2020.12.066. Epub ahead of print. PMID: 33422379.

## Value of the Data

• This is a large data set (n = 3140) of children under 2 years age from a low income, rural south Asian setting from where data on pneumococcal epidemiology is limited.
• The dataset and accompanying files can be used by other researchers interested in the field of pneumococcal epidemiology, public health scientists and mathematical modellers.
• Data can be used to replicate the current analysis, perform same analysis under varying set of assumptions, carry additional exploratory analysis, mathematical modelling and individual level meta-analysis.

## 1 Data Description

Uploaded dataset includes observation on 3140 children under 2 years of age enrolled from October 2014 to September 2018. There are four files which are uploaded.

1. “Raw_dataset.csv
2. “Codebook.xls”
3. “DIB_questionaire.docx”
4. “Lab_questionnaire.docx”

The first two files are to be read in conjunction. There are a total of 92 fields in the “Raw_dataset.csv”. The field (variable) names, variable label, variable type and codes are provided in in “Codebook.xls”. “DIB_questionaire.docx” and “Lab_questionnaire.docx” are the two questionnaires that were used to collect this data. Section A in “DIB_questionaire.docx” documents the eligibility of the study participants and consent given. Section B collects information on sociodemographic variables, past clinical history, and a brief clinical exam. Section C collects information on vaccination history of the child. “Lab_questionnaire.docx” documents information on time of collection and receipt as well as condition of the samples upon receipt at the Infectious Disease Research Laboratory. Fields q12, q13, q14 and q19 in “Raw_dataset.csv” describe the results of the laboratory analysis.

## 2 Experimental Design, Materials and Methods

### 2.1 Study design and Study Area

This was a time series cross sectional study in which was carried out in two Union Councils (Khyber and Shah Alam Shah Jee Wasi Seekhat) of Matiari in Sindh, Pakistan, with a total population of around 88,739. Matiari is a rural agricultural district in Sindh province located around 180 km from Karachi. Its total population is 0.7 million residing in more than 90,000 households in 1,600 villages (2018 census). The household density is 6.7. Only 33% of the population is literate. This site was chosen because of its rural settings with minimum access to health and a high burden of maternal, neonatal and post neonatal illnesses.

### 2.2 Target population

**Fig. 1:**
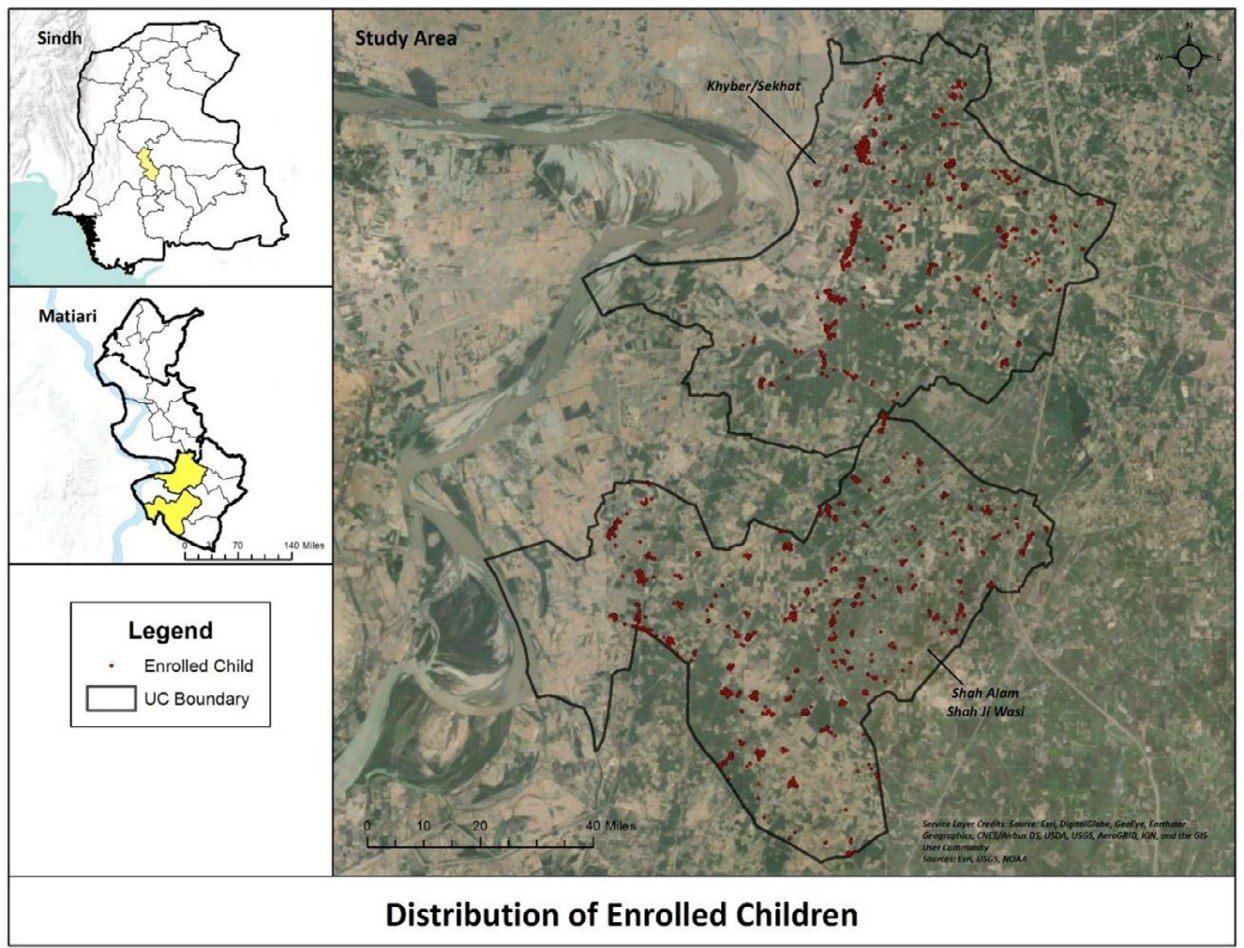
Study site.

Target population was all children under the age of 2 years residing in the study area. Children were randomly selected from a continuously updated line listing every week. Fifteen children who met the inclusion criterion were enrolled per week from October 2014 to September 2018. Children with nose and throat abnormalities or with a serious illness requiring hospitalization were not enrolled. Fig. 1 describes the study area along with GIS coordinates mapped for the enrolled children.

### 2.3 Collection of data

Data was collected by trained study personnel on a custom-made data capturing application for android phones and tablets. Data was collected on household demographics, recent clinical history including hospitalization and outpatient visits, and vaccination status based on caregiver report or vaccination cards wherever available. The online form was created in .xml format using Open Data Kit (ODK) software. To prevent data entry errors, checks were built for consistency, range and logic where applicable.

Nasopharyngeal specimens were collected using established World Health Organization's (WHO) consensus methods and transported at 2–8 °C from the field site to the Infectious Disease Research Laboratory (IDRL) in Karachi within 8 hours of collection [2]. In the lab, samples were vortexed for 10–20 s to disperse the organism and then frozen at -70 °C in an upright position till time of further processing. For culture, samples were thawed, vortexed and 200 µL of a sample was added to a mixture of 1 mL rabbit serum, 5 mL Todd Hewitt broth with 0•5% yeast extract and incubated for 6 h at 37 °C. After this, one loop full (10 ul) was inoculated onto bilayer sheep blood and colistin-nalidixic-acid-agar and streaked for isolation of streptococci. After 18–24 h, plates were examined for the appearance of alpha-hemolytic colonies and susceptibility to optochin and bile solubility. Serotypes were detected using the conventional sequential multiplex PCR assay and further confirmation was done by monoplex PCR [3], [4].

### 2.4 Data storage

Data was stored in a secure server at the Aga Khan University and updated daily. An additional backup was maintained at the Data Monitoring Unit (DMU) of Department of Paediatrics and child Health. Only study investigators and data manager had access to the data.

### 2.5 Data analysis

For the purpose of analysis, epidemiological data was merged with the laboratory data and analysed using Stata version 15 and MS Excel. Variable labels and value labels were created; all categorical variables like gender, illness of past two weeks, vaccination status etc. were presented as frequencies and percentages while continuous variables like age were presented as means and standard deviation. Logistic regression analysis was performed to identify predictors of colonization with a PCV10 serotype. All variables with a p-value less than 0.25 in the bivariate analysis were used to build a multivariable model. A backward selection procedure was used to derive a parsimonious model for retaining only variables significant at *p*-value ≤ 0.05. Unless otherwise indicated, *p* < 0.05 was considered statistically significant.

## Ethics Considerations

Ethical approval was obtained from Aga Khan University's Ethical Review Committee (3181-Ped-ERC-14). Written informed consent from caretakers of the children was obtained before they were enrolled in the study.

## CRediT Author Statement

**Muhammad Imran Nisar:** Conceptualization, Methodology, Formal analysis, Writing - Original Draft, Writing - Review & Editing, Visualization, Supervision, Project administration, Funding acquisition; **Shahira Shahid:** Writing - Original Draft, Writing - Review & Editing, Visualization; **Sajid Muhammad:** Writing - Review & Editing, Data Curation, Software, Formal analysis, Visualization; **Farah Khalid:** Writing - Review & Editing, Data Curation, Software, Formal analysis, Visualization; **Amjad Hussain:** Writing - Review & Editing, Data Curation, Software, Formal analysis, Visualization; **Sheraz Ahmed:** Writing - Review & Editing, Supervision, Project administration; **Sadia Shakoor:** Methodology, Writing - Review & Editing, Investigation; **Furqan Kabir:** Writing - Review & Editing, Investigation, Resources; **Aneeta Hotwani:** Writing - Review & Editing, Investigation, Resources, Supervision; **Asad Ali:** Writing - Review & Editing, Conceptualization, Methodology, Funding acquisition, Supervision; **Anita KM Zaidi:** Writing - Review & Editing, Supervision; **Saad B Omer:** Writing - Review & Editing, Supervision; **Fyezah Jehan:** Conceptualization, Methodology, Writing - Review & Editing, Funding acquisition; **Najeeha Iqbal:** Writing - Review & Editing, Methodology, Formal analysis, Supervision.

## Declaration of Competing Interest

The authors declare that they have no known competing financial interests or personal relationships which have or could be perceived to have influenced the work reported in this article.
